# Isolating the role of elevated *Phlda2* in asymmetric late fetal growth restriction in mice

**DOI:** 10.1242/dmm.017079

**Published:** 2014-08-01

**Authors:** Simon J. Tunster, Mathew Van De Pette, Rosalind M. John

**Affiliations:** Cardiff School of Biosciences, Cardiff University, Cardiff CF10 3AX, UK.

**Keywords:** *Phlda2*, Fetal growth restriction, Asymmetric

## Abstract

Pleckstrin homology-like domain family A member 2 (*PHLDA2*) is a maternally expressed imprinted gene whose elevated expression has been linked to fetal growth restriction in a number of human studies. In mice, *Phlda2* negatively regulates placental growth and limits the accumulation of placental glycogen. We previously reported that a three-copy transgene spanning the *Phlda2* locus drove a fetal growth restriction phenotype late in gestation, suggesting a causative role for *PHLDA2* in human growth restriction. However, in this mouse model, *Phlda2* was overexpressed by fourfold, alongside overexpression of a second imprinted gene, *Slc22a18*. Here, we genetically isolate the role of *Phlda2* in driving late fetal growth restriction in mice. We furthermore show that this *Phlda2*-driven growth restriction is asymmetrical, with a relative sparing of the brain, followed by rapid catch-up growth after birth, classic features of placental insufficiency. Strikingly, fetal growth restriction showed strain-specific differences, being apparent on the 129S2/SvHsd (129) genetic background and absent on the C57BL6 (BL6) background. A key difference between these two strains is the placenta. Specifically, BL6 placentae possess a more extensive endocrine compartment and substantially greater stores of placental glycogen. Taken together, these data support a direct role for elevated *Phlda2* in limiting fetal growth but also suggest that growth restriction only manifests when there is limited placental reserve. These findings should be taken into account in interpreting the results from human studies.

## INTRODUCTION

Low birth weight (LBW) is one of the most problematic conditions affecting human populations. It is a very common complication of pregnancy, affecting up to 19% of all births in the developing world and between 5 and 7% of births in developed countries (Valero de Bernabe et al., 2004). Either as a consequence of poor growth *in utero*, preterm birth or a genetic abnormality, some babies are born too small and are consequently more vulnerable to complications at the time of birth, with increased risk of mortality (Witt et al., 2012). Furthermore, it is increasingly recognised that, although these vulnerable babies can gain weight and seem to recover from their poor start in life, many will experience complications in later life, including alterations in behaviour (ADHD, depression) and metabolism (obesity, type 2 diabetes, heart disease) (Grissom and Reyes, 2013). Identifying the molecular origins of growth restriction is crucial in identifying the most appropriate management of LBW infants to ensure optimal short- and long-term health outcomes.

Genomic imprinting is an epigenetic phenomenon that drives the preferential expression of certain genes from one parental allele (John and Surani, 1996). In humans, a number of childhood growth disorders, including Beckwith-Wiedemann syndrome (BWS; MIM #130650) and Silver-Russell syndrome (SRS; MIM #180860), involve the aberrant expression of imprinted genes (Chiesa et al., 2012). These imprinting disorders are rare at 1 in 13,000 and 1 in 300,000 births, respectively. However, numerous imprinted genes play a key role in regulating fetal growth and placental development in a dosage-sensitive manner, with paternal silencing primarily of growth-restricting genes and maternal silencing of growth-promoting genes (Tunster et al., 2013), which suggests that aberrant imprinting might underlie more common human growth disorders.

Pleckstrin homology-like domain family A member 2 (*PHLDA2*) was first highlighted as a potential fetal growth restriction gene in a 2006 study that reported elevated expression in the placenta of 9 out of the 38 fetal growth restriction (FGR) placentae (McMinn et al., 2006). Subsequently, several studies have examined *PHLDA2* in relation to fetal growth and birth weight (Jensen et al., 2014). Whereas some of these studies have supported a role for elevated *PHLDA2* in LBW (Apostolidou et al., 2007; Guo et al., 2008; Lim et al., 2012), others did not find a clear correlation with birth weight (Lambertini et al., 2012; Lewis et al., 2012).

We previously reported on a growth restriction phenotype in mice that was linked to the presence of three copies of a bacterial artificial chromosome (BAC) spanning the murine *Phlda2* locus and a second imprinted gene, solute carrier family 22, member 18 (*Slc22a18*) (Salas et al., 2004; Tunster et al., 2010). Transgenic fetuses were 13% lighter than control littermates late in gestation, with significant placental stunting phenotype and a severe loss of placental glycogen. However, these studies did not establish a direct link between elevated *Phlda2* expression and fetal growth restriction, nor did they model the more subtle elevations in expression reported in human studies. Here, we sought to clarify the role of the *Phlda2* gene in regulating fetal growth by using transgenic mice carrying a single-copy transgene. We report that as little as twofold increased expression of *Phlda2* was sufficient to reduce birth weight by 10%. Importantly, growth restriction was asymmetric, with relative sparing of the brain, followed by rapid catch-up growth after birth, classic features of placental insufficiency.

## RESULTS

**Fig. 1.**
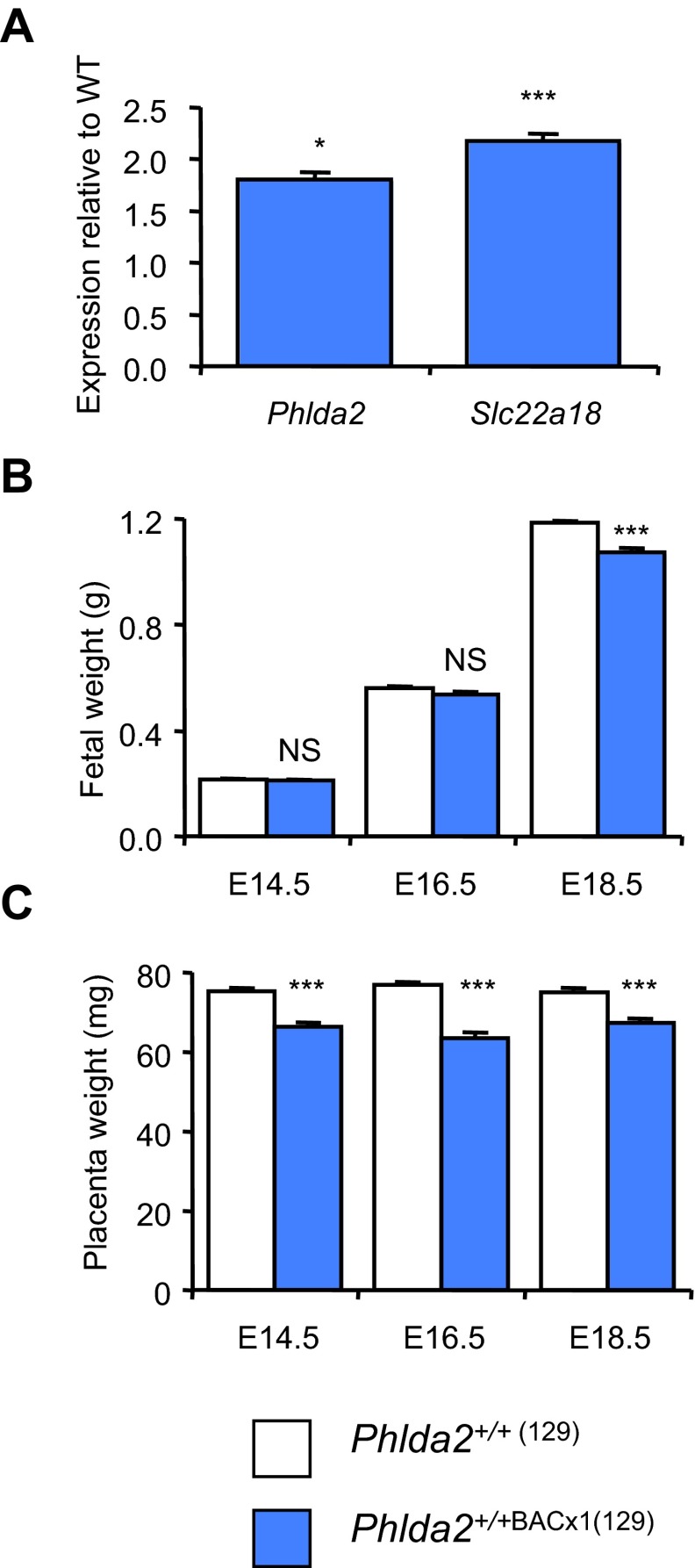
**Characterisation of the single-copy line BACx1 on a 129 strain background.** (A) QPCR analysis of *Phlda2* and *Slc22a18* expression in E14.5 *Phlda2*^+/+BACx1(129)^ placentae. (B) Fetal wet weights on the 129 genetic background at E14.5, E16.5 and E18.5. *Phlda2*^+/+BACx1(129)^ (2×) fetuses (*n*=36) were significantly lighter at E18.5, weighing 90.7% of the weight of their non-transgenic counterparts (*P*=5.33×10^−6^). (C) Placental wet weights on the 129 genetic background. *Phlda2*^+/+BACx1(129)^ (2×) placentae were significantly lighter that non-transgenic placentae at each time point. Numerical data is given in supplementary material Table S1. NS, not significant (*P*>0.05), *P<0.05, ***P<0.005.

In our previous paper, gene expression analysis of a newly generated BAC transgenic line on mixed genetic background suggested the presence of at least two copies of the BAC transgene (Tunster et al., 2010). However, when we bred this line fully into the 129S2/SvHsd (129) genetic background (>eight generations) and re-examined gene expression, the data was more consistent with the presence of a single copy (Fig. 1A). When we examined fetal weight in the context of twofold gene expression, growth restriction of *Phlda2*^+/+BACx1^ fetuses was apparent at embryonic day 18.5 (E18.5; 1184.4±8.7 mg versus 1073.8±18.5 mg, *P*=5.33×10^−6^) but not at E14.5 or E16.5 (Fig. 1B), similar to the growth profile we reported in response to fourfold expression (Tunster et al., 2010). Transgenic placentae were 10–20% lighter than non-transgenic placentae at each time point (Fig. 1C), similar to the weight reduction we reported for a single-copy transgenic line spanning *Phlda2*, *Slc22a18* and *Cdkn1c* (Tunster et al., 2010).

**TRANSLATIONAL IMPACT**

**Clinical issue**

Low birth weight is one of the most intractable and yet clinically relevant complications affecting human pregnancies. Being born small increases the risk of complications at the time of birth and can lead to early mortality. In addition, although low-birth-weight babies often gain weight and seem to recover from their poor start in life, babies with accelerated postnatal weight gain after *in utero* growth restriction have a greatly increased risk of developing type 2 diabetes, obesity and cardiovascular disease in later life compared with normal-birth-weight babies. Although the causes of low birth weight are numerous and include both genetic and environmental factors, elevated placental expression of the imprinted gene *PHLDA2* has been reported in a number of studies on low birth weight. However, because correlation does not always equal causation, determination of the relevance of elevated *PHLDA2* expression using an animal model is of crucial importance.

**Results**

In this study, the authors generated a mouse model in which *Phlda2* expression was elevated twofold, a similar increase in expression to that reported in human studies. The authors observed late fetal growth restriction in the transgenic mice, with the pups being born with a low birth weight. Crucially, growth restriction was asymmetric, with relative sparing of the brain, followed by rapid catch-up growth after birth, which are classic features of placental insufficiency. Moreover, growth restriction showed strain-specific differences and the effects of *Phlda2* overexpression were only apparent in mouse strains in which the placenta was already working at maximum capacity.

**Implications and future directions**

These findings support a causal role for elevated *PHLDA2* in the aetiology of human low birth weight. Moreover, the observation of both asymmetrical growth restriction and rapid catch-up growth in this new mouse model of *Phlda2* overexpression suggests that human infants born with a low birth weight owing to this specific alteration might be at higher risk of later-life health complications than infants born with a low birth weight for other reasons. This possibility can be investigated by reexamining data from human cohorts and through further work on the animal model. Finally, these findings suggest that elevated *PHLDA2* expression might be most harmful in scenarios in which the placenta is working at maximum capacity, such as in pregnancies subject to prenatal adversity.

**Fig. 2.**
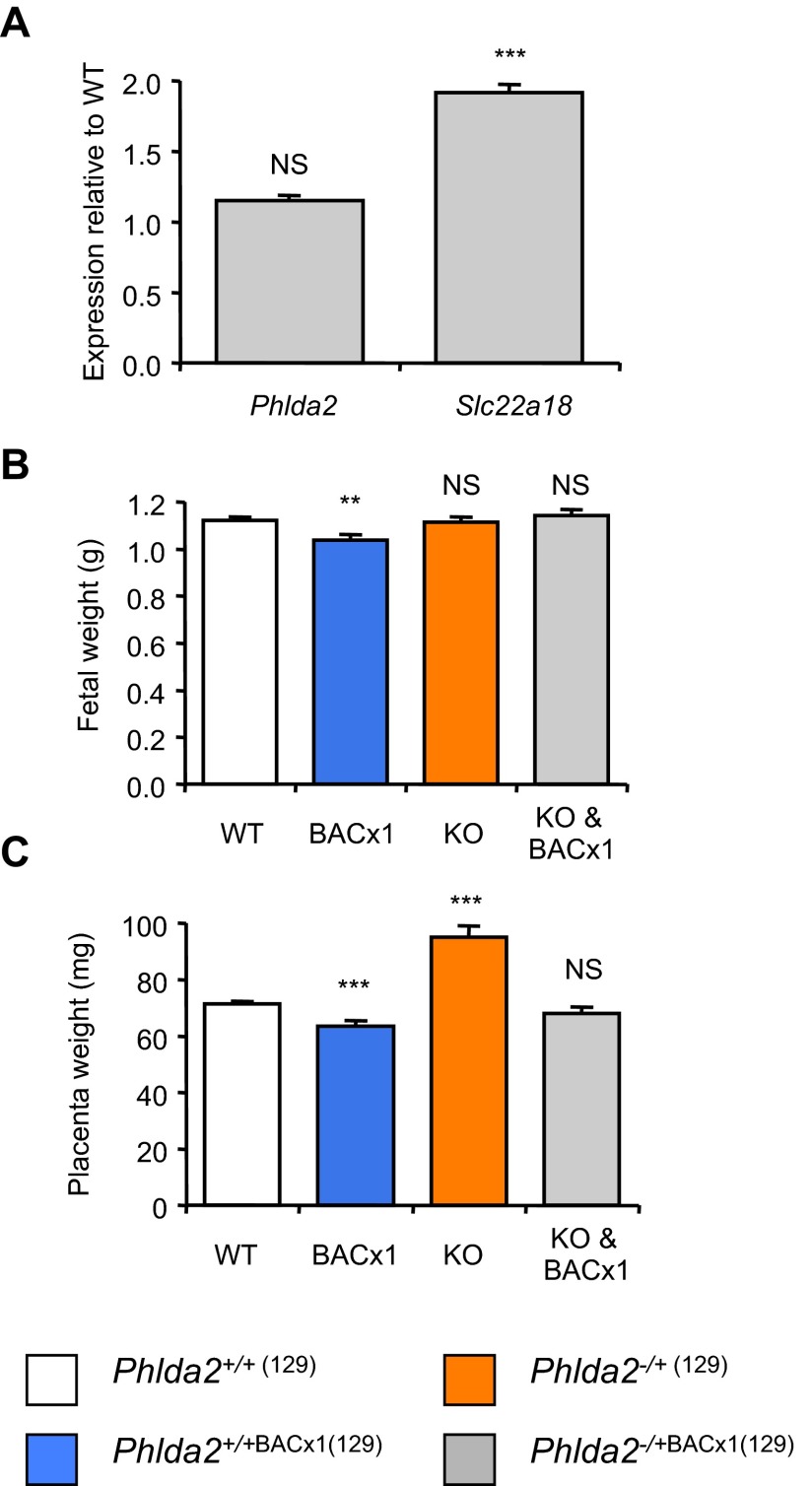
**Isolating a causative role for elevated *Phlda2* in inducing late fetal growth restriction (129 strain background).** (A) QPCR analysis of *Phlda2* and *Slc22a18* expression in E14.5 *Phlda2*^+/+^*^(129)^* (wild type; non-transgenic) versus *Phlda2*^−/+BACx1(129)^ (1×) (double transgenic) from litters containing all four genotypes. *Phlda2* expression was normal in double-transgenic placenta. (B) E18.5 fetal wet weights for *Phlda2*^+/+^*^(129)^* (WT), *Phlda2*^+/+BACx1(129)^ (BACx1; 2×), *Phlda2*^−/+(129)^ (KO; 0×) and *Phlda2*^−/+BACx1(129)^ (KO & BACx1; 1×) from litters containing all four genotypes on the 129 genetic background. Fetal growth restriction was attenuated in double-transgenic fetuses. *Phlda2*^−/+(129)^ (0×) fetuses were similar in weight to controls. (C) E18.5 placental wet weights for the litters used to generate data in Fig. 2B. Placental growth restriction was apparent only when *Phlda2* expression was elevated (*Phlda2*^+/+BACx1(129)^). *Phlda2*^−/+(129)^ (0×) placentae were 33% heavier than controls. Numerical data is given in supplementary material Table S2. NS, not significant (*P*>0.05), **P<0.01, ***P<0.005.

To formally test whether the line carried a single copy of the transgene, we crossed males carrying the BAC with females carrying a paternally inherited targeted allele of *Phlda2*, also bred for >eight generations into the 129 genetic background. This cross generated four genotypes: *Phlda2*^+/+(129)^ (one active copy of *Phlda2* and *Slc22a18*), *Phlda2*^+/+BACx1(129)^ (two active copies *Phlda2* and *Slc22a18*), *Phlda2*^−/+(129)^ (maternal-knockout *Phlda2*, equivalent to loss of function) and *Phlda2*^−/+BACx1(129)^ (double transgenic; one active copy of *Phlda2* and two active copies of *Slc22a18*). At E14.5, *Phlda2* was expressed at wild-type levels in double-transgenic fetuses, whereas *Slc22a18* remained elevated, confirming that the line carried a single extra copy of *Phlda2* (Fig. 2A). Importantly, these double-transgenic fetuses exposed to a single dose of *Phlda2* and a double dose of *Slc22a18* were not significantly different in weight to controls at E18.5 (1123.3±13.8 mg versus 1144.3±23.4 mg, *P*=0.415; Fig. 2B), whereas fetuses carrying the single-copy BAC transgene, exposed to a double dose of both genes (*Phlda2*^+/+BACx1(129)^), weighed less than controls (1123.3±13.8 mg versus 1038.7±23.4 mg, *P*=0.00180; Fig. 2B). This finding genetically assigned growth restriction to the twofold-elevated *Phlda2* expression. As observed with a different single-copy BAC transgene (Tunster et al., 2010), normalising the dose of *Phlda2* also returned placental weights to normal (Fig. 2C).

Loss of function of *Phlda2* has previously been characterised on the C57BL/6 genetic background (Frank et al., 2002; Salas et al., 2004). We similarly observed substantial placental overgrowth on the 129 background. *Phlda2*^−/+(129)^ placentae weighed 33% more than control placentae at E18.5 (94.9±4.1 mg versus 71.5±0.9 mg; *P*=8.41×10^−8^; Fig. 2C). As in the study of *Phlda2* loss of function on the BL6 background, *Phlda2*^−/+(129)^ fetuses, which lacked the maternal *Phlda2* allele, were not advantaged by the possession of a larger placenta and weighed the same as their non-transgenic counterparts at E18.5 (1116.1±19.1 mg versus 1123.3±13.8; *P*=0.753; Fig. 2B). These data formally assigned the late fetal growth restriction phenotype to twofold-elevated expression of *Phlda2* and confirmed that loss of function of *Phlda2* does not induce fetal overgrowth.

**Fig. 3.**
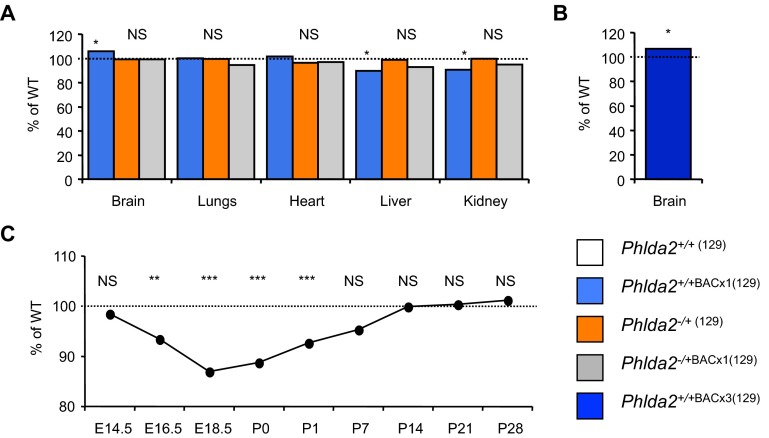
***Phlda2*-induced fetal growth restriction is asymmetric (129 strain background).** (A) Ratio of brain, lungs, heart, liver and kidney wet weights at E18.5 expressed as a proportion of body weight taken from samples described in Fig. 2. Brain weight was heavier as a proportion of body weight, compared with controls, whereas liver and kidney, sites of embryonic *Phlda2* expression, were lighter. Normalising *Phlda2* expression restored organ weight to non-transgenic proportions. (B) Ratio of brain as a proportion of body weight in E18.5 fetuses carrying three copies of the BAC transgene, *Phlda2*^+/+BACx3(129)^ (4×). (C) Fetal and postnatal weight data for the line *Phlda2*^+/+BACx3(129)^ (4×). Transgenic fetuses were lighter at birth but rapidly gained weight such that there was no significant difference in weight to their non-transgenic counterparts by P7. Numerical data is given in supplementary material Table S3. NS, not significant (*P*>0.05), *P<0.05, **P<0.01, ***P<0.005.

Fetal growth restriction due to placental insufficiency is often asymmetric, with a relative reduction in fetal kidney, liver, pancreas and lung size but a sparing of the brain. When organ weights of the four genotypes were compared at E18.5, the brain weights of the *Phlda2*^+/+BACx1^ fetuses were 10% heavier relative to body weight as compared to the non-transgenic fetuses, suggestive of brain sparing (Fig. 3A). The lungs and heart were proportionately growth restricted, whereas the liver and kidney, sites of embryonic *Phlda2* expression (Qian et al., 1997), were growth restricted as a proportion of body weight (Fig. 3A). Brain sparing was similarly apparent in newborn mice carrying three copies of the transgene (*Phlda2*^+/+BACx3^) examined on the 129 genetic background (Fig. 3B). Normalising the dose of *Phlda2* restored symmetry to the fetus (Fig. 3A). These data genetically demonstrated that twofold elevated *Phlda2* restricts fetal growth asymmetrically. Moreover, loss of function of *Phlda2* had no significant consequence for these organ weights, excluding a reciprocal function for *Phlda2* in regulating organ weights.

Catch-up growth is a key feature of extrinsically driven fetal growth restriction (Saenger et al., 2007). When postnatal weights for line *Phlda2*^+/+BACx3^ were examined from birth (P0) until 4 weeks of age (P28), pups were born weighing 11% less than their non-transgenic counterparts (1.25±0.014 g versus 1.41±0.009 g; *P*=3.29×10^−17^). Within 7 days, there was no significant difference in the weights between transgenic and non-transgenic pups (*P*=0.147; Fig. 3C).

**Fig. 4.**
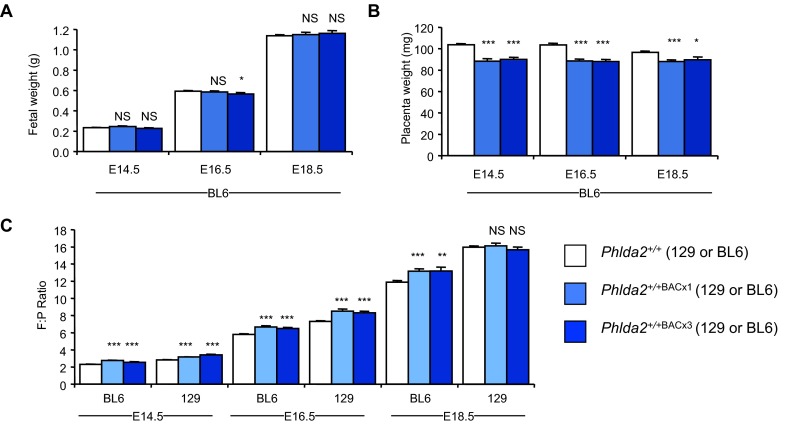
**Absence of fetal growth restriction on the BL6 genetic background.** (A) Fetal wet weights on the BL6 genetic background at E14.5, E16.5 and E18.5 for *Phlda2*^+/+BACx1(BL6)^ (2×), and at E18.5 for *Phlda2*^+/+BACx3(BL6)^ (4×). Fetuses were the same weight as their non-transgenic counterparts at each time point. (B) Placental wet weights on the BL6 genetic background at E14.5, E16.5 and E18.5 for *Phlda2*^+/+BACx1(BL6)^ (2×) and at E18.5 for *Phlda2*^+/+BACx3(BL6)^ (4×). Placentae were significantly lighter that non-transgenic placentae at each time point. (C) Fetal:placental (F:P) weight ratios. Ratios were significantly different compared with non-transgenic mice at each time point on the BL6 genetic background, but only at E14.5 and E16.5 on the 129 genetic background. Numerical data is given in supplementary material Table S4. NS, not significant (*P*>0.05), *P<0.05, **P<0.01, ***P<0.005.

Placental weight and fetal:placental (F:P) ratios are widely used as a parameter of placental functional capacity (Fowden et al., 2009). The F:P ratio for mice of the 129 genetic background at E18.5 is relatively high, at 16.0±0.23, whereas that of C57BL/6 (BL6) mice is lower, at 12.3±0.18 (Tunster et al., 2012). This suggests that BL6 placenta might have a greater reserve capacity than 129 placenta. To investigate whether *Phlda2* similarly restrained fetal growth in the context of a more favourable F:P ratio, we bred *Phlda2*^+/+BACx1^ and *Phlda2*^+/+BACx3^ (three copy line) mice onto the BL6 genetic background for >eight generations. We observed no difference in fetal weight at E14.5, E16.5 or E18.5 (Fig. 4A) despite transgenic placentae weighing 10–15% less than non-transgenic placentae at each time point (Fig. 4B). When we examined F:P ratios, there was a significant difference from normal at every time point due to the reduction in weight of the placenta but not the fetus on the BL6 background (Fig. 4C). F:P ratios were also significantly different from normal at E14.5 and E16.5 on the 129 background. At E18.5, there was no significant difference in F:P ratio because, at this later time point, both placental and fetal weights were reduced. This suggests that 129 placentae function at maximum capacity late in gestation and consequently cannot compensate for the reduction in capacity induced by elevated *Phlda2*, resulting in fetal growth restriction

**Fig. 5.**
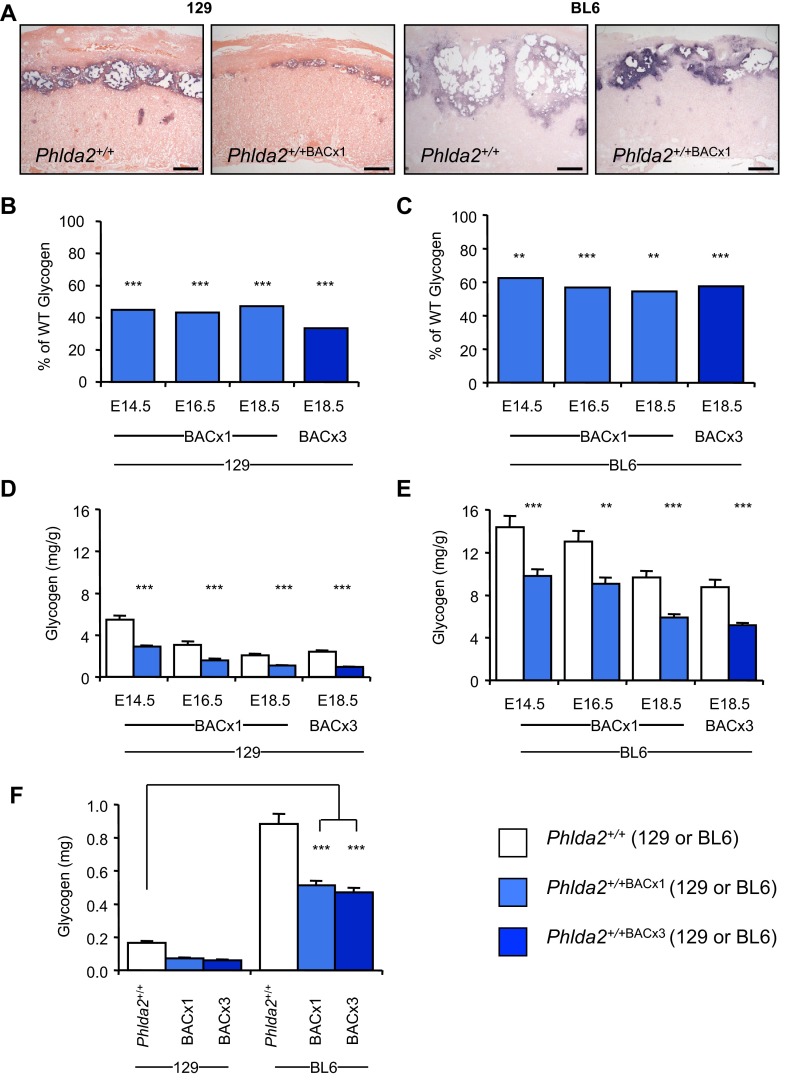
**Placental glycogen attenuates fetal growth restriction.** (A) *In situ* hybridisation of *Tpbpa* riboprobe to E14.5 sections from *Phlda2*^+/+(129)^ and *Phlda2*^+/+BACx1(129)^ (2×) placenta in comparison to the same genotypes on the BL6 genetic background. Scale bars: 200 μm. (B) Biochemical determination of glycogen at E14.5, E16.5 and E18.5 for *Phlda2*^+/+BACx1^ and at E18.5 for *Phlda2*^+/+BACx3^ (4×) on the 129 background expressed as a percentage of values in non-transgenic placenta. (C) Biochemical determination of glycogen at E14.5, E16.5 and E18.5 for *Phlda2*^+/+BACx1^ and at E18.5 for *Phlda2*^+/+BACx3^ on the BL6 background expressed as a percentage of values in control placenta. (D) 129 glycogen data from panel B expressed as mg/g of placenta. (E) BL6 glycogen data from panel C expressed as mg/g of placenta. (F) Comparison of total glycogen stored in E18.5 placenta on the 129 versus BL6 background. Compromised (*Phlda2* overexpression) BL6 placentae contain three times more glycogen than that found in the uncompromised (*Phlda2* wild-type expression) 129 placentae. Numerical data is given in supplementary material Table S5. **P<0.01, ***P<0.005.

A reduced junctional zone and loss of placental glycogen have been linked to fetal growth restriction in several studies (Hitz et al., 2005; Oh-McGinnis et al., 2011; Withington et al., 2006; Zheng-Fischhöfer et al., 2007). Furthermore, placental glycogen has been suggested as an important source of easily utilisable energy to support late embryonic growth (Coan et al., 2006). In addition to the difference in F:P ratios, BL6 placentae have a disproportionately larger junctional zone and significantly greater stores of placental glycogen than 129 placentae (Tunster et al., 2012). *In situ* hybridisation with trophoblast specific protein alpha (*Tpbpa*), a marker for the junctional zone (Lescisin et al., 1988), revealed a reduction of the junctional zone at E14.5 on both the 129 and the BL6 genetic backgrounds for line *Phlda2*^+/+BACx1^ (Fig. 5A). To establish to what extent elevated *Phlda2* compromised glycogen accumulation on the two genetic backgrounds, a biochemical determination of glycogen was performed at E14.5, E16.5 and E18.5. Twofold expression of *Phlda2* induced a 50–60% decrease in total glycogen on the 129 background and 40–45% decrease on the BL6 background, relative to the respective non-transgenic counterparts, at each time point (Fig. 5B,C). A similar reduction was apparent for the three-copy line at E18.5 on both backgrounds (Fig. 5B,C). Thus, elevated *Phlda2* similarly limited the accumulation of glycogen on both genetic backgrounds. However, although the amplitude of the effect was similar, a comparison of the amount of glycogen stored, expressed either relative to the weight of the placenta (mg/g) or as a total amount (mg), highlighted a striking finding. Whereas glycogen stores were compromised on both genetic backgrounds by elevated *Phlda2*, the compromised BL6 placentae (*Phlda2*^+/+BACx1(BL6)^) still contained three times more glycogen than the genetically uncompromised 129 placentae (*Phlda2*^+/+(129)^) (Fig. 5D–F).

Taken together, these data genetically define a role for elevated *Phlda2* in asymmetrically restricting late fetal growth but suggests that the growth-restricting properties of *Phlda2* manifest only in unfavourable circumstances in which the placenta is already functioning at its upper limit to support the growth demands of the rapidly growing fetus.

## DISCUSSION

Elevated expression of the imprinted gene *PHLDA2* has been reported in a number of studies on LBW and fetal growth restriction, but correlation does not always equal causation. Here, we have demonstrated, using a mouse model, that twofold elevated expression of *Phlda2* can restrain fetal growth late in gestation. Moreover, we have also shown that *Phlda2*-induced growth restriction is asymmetric, with a relative sparing of the brain alongside rapid postnatal catch-up growth. However, we did not observe a similar growth restriction on a different genetic background.

Mouse and human placentae differ substantially not only with respect to histologically classified lineages but also with respect to gene expression (Carter, 2012). Nonetheless, our data support a causal role for elevated *PHLDA2* in restricting fetal growth in human pregnancies. In the study by McMinn et al., ~25% of the placentae from growth-restricted babies showed increased expression of *PHLDA2* (McMinn et al., 2006). Kumar et al. similarly reported elevated placental *PHLDA2* in relation to fetal growth restriction, and Diplas et al. reported a threefold increase in placental *PHLDA2* in very LBW babies (Kumar et al., 2012; Diplas et al., 2009). These data suggest that elevated *PHLDA2* might be a relatively common cause of LBW in human populations. Our findings in the mouse model that *Phlda2*-induced growth restriction was asymmetric, with relative sparing of the brain, followed by rapid catch-up growth, is consequently of significant importance. Human babies with these characteristics, particularly those with accelerated postnatal weight gain after *in utero* growth restriction, have a greatly enhanced risk of developing type 2 diabetes, obesity and cardiovascular disease [Barker, 1990; Barker, 1994; Barker, 2004; Barker et al., 2006; Barker et al., 1991; Barker, 2001; Barker and Medical Research Council, Environmental Epidemiology Unit (UK), 1992; Lackland et al., 2000; Lackland et al., 2001]. These diseases are thought to reflect the programming of metabolic abnormalities *in utero* in response to limited nutrient supply acerbated by optimal or even excessive nutrition after birth. This suggests that babies with elevated placental *PHLDA2* might be at risk of these later-life health complications. Further work is required to follow up the later-life outcomes for babies with elevated *PHLDA2* and also our transgenic mice to identify the predictive value of *PHLDA2* as a biomarker.

Several human studies suggest that *PHLDA2* expression inversely correlates with birth weight even within normal pregnancies (Apostolidou et al., 2007; Guo et al., 2008; Lim et al., 2012). Moreover, in a study of >7000 samples over three cohorts, a *PHLDA2* promoter polymorphism was positively correlated with birth weight (Ishida et al., 2012). An inverse correlation with LBW was not reproduced in two other studies (Lambertini et al., 2012; Lewis et al., 2012), although these studies only looked at ~100 samples. In our previous investigation of *PHLDA2* expression in placentae from the Southampton Women’s Study, we did not find a significant inverse association between placental *PHLDA2* and birth weight, but we did observe a slowdown in fetal femur growth late in gestation and a significant loss of bone density at age 4 years (Lewis et al., 2012). In this current study, we found that the growth-restricting properties of *Phlda2* were apparent on the 129 and not the BL6 genetic background, despite similar proportional reductions in placental weight and glycogen stores. Both the human data and our mouse data can be reconciled if elevated *PHLDA2* only restricts fetal growth under certain circumstances, i.e. when the placenta is working at maximum capacity or when other factors, such as environmental exposures, limit placental capacity extrinsically. LBW is particularly prevalent in populations under severe socioeconomic stress; this could be due to a multitude of factors including poor diet, maternal stress and smoking. Human placental *PHLDA2* has been reported to be upregulated in response to maternal smoking; additionally, in an experimental rat model, both maternal calorie restriction and alcohol were found to result in significantly upregulated placental *Phlda2* (Bruchova et al., 2010; Shukla et al., 2011). We did not identify a correlation between placental *PHLDA2* expression and maternal lifestyle in our relatively small study (Lewis et al., 2012), but it might be pertinent to further explore the data on *PHLDA2* both with respect to asymmetry and catch-up growth while taking into account the degree of prenatal adversity experienced by the mother.

In summary, our work has identified a causative role for elevated *Phlda2* in inducing asymmetrical late fetal growth restriction. Importantly, our work also suggests that elevated *Phlda2* might be more harmful in scenarios where the placenta is working at maximum capacity, such as in pregnancies subject to prenatal adversity.

## MATERIALS AND METHODS

### Mouse strains and genotyping

Animal studies and breeding were approved by the Universities of Cardiff ethical committee and performed under a UK Home Office project licence (RMJ). All mice were housed together in one room throughout the study on a 12-hour light-dark cycle with lights coming on at 06.00 hour and a temperature range of 21±2°C with free access to water (tap water) and standard chow. The *Phlda2* transgenic lines *Phlda2*^+/+BACx1(129)^ (previously Tg^10–10^) and *Phlda2*^+/+BACx3(129)^ (previously Tg^10–15^) were bred for >eight generations onto either the C57BL6 (Harlan, BL6) or the 129S2/SvHsd (Harlan, 129) strain backgrounds for phenotypic assessment. The *Phlda2* targeted allele was either maintained by paternal transmission on the BL6 background or crossed into the 129 background for >eight generations.

### Weighing studies and biochemical determination of placental glycogen concentration

Fetal and placental wet weights were taken at the stated time points after a discernible plug. Genotyping data was obtained from yolk sac DNA as previously described (Frank et al., 2002; John et al., 2001). Glycogen was extracted from whole placenta, and resuspended in 1 ml of H_2_O and assayed according to the method of Lo et al. (Lo et al., 1970) at a dilution of 1 in 2 (129) or 1 in 10 (BL6).

### Quantitative RNA analysis

Quantitative PCR of reverse-transcribed RNA (QRT-PCR) was performed and analysed as described (Schmittgen and Livak, 2008; Tunster et al., 2010).

### Statistical analyses

Statistical significance (probability values) was determined using the Student’s *t*-test (two-tailed distribution and two-sample unequal variance) or the Mann-Whitney *U*-test.

## Supplementary Material

Supplementary Material

## References

[b1-0071185] Apostolidou S. Abu-Amero S. O’Donoghue K. Frost J. Olafsdottir O. Chavele K. M. Whittaker J. C. Loughna P. Stanier P. Moore G. E. (2007). Elevated placental expression of the imprinted PHLDA2 gene is associated with low birth weight. J. Mol. Med. 85, 379–387 17180344 10.1007/s00109-006-0131-8

[b2-0071185] Barker D. J. (1990). The fetal and infant origins of adult disease. *BMJ* 301, 1111. 10.1136/bmj.301.6761.1111 PMC1664286 2252919

[b3-0071185] Barker D. J. (1994). Maternal and fetal origins of coronary heart disease. J. R. Coll. Physicians Lond. 28, 544–551 7884713 PMC5401118

[b4-0071185] Barker D. J. P. (2001). Fetal Origins of Cardiovascular and Lung Disease. New York, NY: Marcel Dekker

[b5-0071185] Barker D. J. (2004). The developmental origins of well-being. Philos. Trans. R. Soc. B 359, 1359–1366 10.1098/rstb.2004.1518 PMC1693427 15347527

[b6-0071185] Barker D. J. P. Medical Research Council, Environmental Epidemiology Unit (UK) (1992). Fetal and Infant Origins of Adult Disease: Papers. London: British Medical Journal

[b7-0071185] Barker D. J. Godfrey K. M. Fall C. Osmond C. Winter P. D. Shaheen S. O. (1991). Relation of birth weight and childhood respiratory infection to adult lung function and death from chronic obstructive airways disease. BMJ 303, 671–675 1912913 10.1136/bmj.303.6804.671 PMC1670943

[b8-0071185] Barker D. J. Bagby S. P. Hanson M. A. (2006). Mechanisms of disease: in utero programming in the pathogenesis of hypertension. Nat. Clin. Pract. Nephrol. 2, 700–707 17124527 10.1038/ncpneph0344

[b9-0071185] Bruchova H. Vasikova A. Merkerova M. Milcova A. Topinka J. Balascak I. Pastorkova A. Sram R. J. Brdicka R. (2010). Effect of maternal tobacco smoke exposure on the placental transcriptome. Placenta 31, 186–191 20092892 10.1016/j.placenta.2009.12.016

[b10-0071185] Carter A. M. (2012). Evolution of placental function in mammals: the molecular basis of gas and nutrient transfer, hormone secretion, and immune responses. Physiol. Rev. 92, 1543–1576 23073626 10.1152/physrev.00040.2011

[b11-0071185] Chiesa N. De Crescenzo A. Mishra K. Perone L. Carella M. Palumbo O. Mussa A. Sparago A. Cerrato F. Russo S. (2012). The KCNQ1OT1 imprinting control region and non-coding RNA: new properties derived from the study of Beckwith-Wiedemann syndrome and Silver-Russell syndrome cases. Hum. Mol. Genet. 21, 10–25 21920939 10.1093/hmg/ddr419 PMC3235007

[b12-0071185] Coan P. M. Conroy N. Burton G. J. Ferguson-Smith A. C. (2006). Origin and characteristics of glycogen cells in the developing murine placenta. Dev. Dyn. 235, 3280–3294 17039549 10.1002/dvdy.20981

[b13-0071185] Diplas A. I. Lambertini L. Lee M. J. Sperling R. Lee Y. L. Wetmur J. Chen J. (2009). Differential expression of imprinted genes in normal and IUGR human placentas. Epigenetics 4, 235–240 19483473 10.4161/epi.9019

[b14-0071185] Fowden A. L. Sferruzzi-Perri A. N. Coan P. M. Constancia M. Burton G. J. (2009). Placental efficiency and adaptation: endocrine regulation. J. Physiol. 587, 3459–3472 19451204 10.1113/jphysiol.2009.173013 PMC2742275

[b15-0071185] Frank D. Fortino W. Clark L. Musalo R. Wang W. Saxena A. Li C. M. Reik W. Ludwig T. Tycko B. (2002). Placental overgrowth in mice lacking the imprinted gene Ipl. Proc. Natl. Acad. Sci. USA 99, 7490–7495 12032310 10.1073/pnas.122039999 PMC124258

[b16-0071185] Grissom N. M. Reyes T. M. (2013). Gestational overgrowth and undergrowth affect neurodevelopment: similarities and differences from behavior to epigenetics. Int. J. Dev. Neurosci. 31, 406–414 23201144 10.1016/j.ijdevneu.2012.11.006 PMC6288817

[b17-0071185] Guo L. Choufani S. Ferreira J. Smith A. Chitayat D. Shuman C. Uxa R. Keating S. Kingdom J. Weksberg R. (2008). Altered gene expression and methylation of the human chromosome 11 imprinted region in small for gestational age (SGA) placentae. Dev. Biol. 320, 79–91 18550048 10.1016/j.ydbio.2008.04.025

[b18-0071185] Hitz C. Vogt-Weisenhorn D. Ruiz P. Wurst W. Floss T. (2005). Progressive loss of the spongiotrophoblast layer of Birc6/Bruce mutants results in embryonic lethality. Genesis 42, 91–103 15887267 10.1002/gene.20128

[b19-0071185] Ishida M. Monk D. Duncan A. J. Abu-Amero S. Chong J. Ring S. M. Pembrey M. E. Hindmarsh P. C. Whittaker J. C. Stanier P. (2012). Maternal inheritance of a promoter variant in the imprinted PHLDA2 gene significantly increases birth weight. Am. J. Hum. Genet. 90, 715–719 22444668 10.1016/j.ajhg.2012.02.021 PMC3322226

[b20-0071185] Jensen A. B. Tunster S. J. John R. M. (2014). The significance of elevated placental PHLDA2 in human growth restricted pregnancies. Placenta 35, 528–532 24953163 10.1016/j.placenta.2014.04.018

[b21-0071185] John R. M. Surani M. A. (1996). Imprinted genes and regulation of gene expression by epigenetic inheritance. Curr. Opin. Cell Biol. 8, 348–353 8743885 10.1016/s0955-0674(96)80008-1

[b22-0071185] John R. M. Ainscough J. F. Barton S. C. Surani M. A. (2001). Distant cis-elements regulate imprinted expression of the mouse p57(Kip2) (Cdkn1c) gene: implications for the human disorder, Beckwith–Wiedemann syndrome. Hum. Mol. Genet. 10, 1601–1609 11468278 10.1093/hmg/10.15.1601

[b23-0071185] Kumar N. Leverence J. Bick D. Sampath V. (2012). Ontogeny of growth-regulating genes in the placenta. Placenta 33, 94–99 22154689 10.1016/j.placenta.2011.11.018

[b24-0071185] Lackland D. T. Bendall H. E. Osmond C. Egan B. M. Barker D. J. (2000). Low birth weights contribute to high rates of early-onset chronic renal failure in the Southeastern United States. Arch. Intern. Med. 160, 1472–1476 10826460 10.1001/archinte.160.10.1472

[b25-0071185] Lackland D. T. Egan B. M. Fan Z. J. Syddall H. E. (2001). Low birth weight contributes to the excess prevalence of end-stage renal disease in African Americans. J. Clin. Hypertens. (Greenwich) 3, 29–31 11416679 10.1111/j.1524-6175.2001.00828.x PMC8101806

[b26-0071185] Lambertini L. Marsit C. J. Sharma P. Maccani M. Ma Y. Hu J. Chen J. (2012). Imprinted gene expression in fetal growth and development. Placenta 33, 480–486 22465419 10.1016/j.placenta.2012.03.001 PMC3348252

[b27-0071185] Lescisin K. R. Varmuza S. Rossant J. (1988). Isolation and characterization of a novel trophoblast-specific cDNA in the mouse. Genes Dev. 2 12A, 1639–1646 10.1101/gad.2.12a.1639 3215514

[b28-0071185] Lewis R. M. Cleal J. K. Ntani G. Crozier S. R. Mahon P. A. Robinson S. M. Harvey N. C. Cooper C. Inskip H. M. Godfrey K. M. Southampton Women’s Survey Study Group (2012). Relationship between placental expression of the imprinted PHLDA2 gene, intrauterine skeletal growth and childhood bone mass. Bone 50, 337–342 22100507 10.1016/j.bone.2011.11.003 PMC3657144

[b29-0071185] Lim A. L. Ng S. Leow S. C. Choo R. Ito M. Chan Y. H. Goh S. K. Tng E. Kwek K. Chong Y. S. (2012). Epigenetic state and expression of imprinted genes in umbilical cord correlates with growth parameters in human pregnancy. J. Med. Genet. 49, 689–697 23042810 10.1136/jmedgenet-2012-100858

[b30-0071185] Lo S. Russell J. C. Taylor A. W. (1970). Determination of glycogen in small tissue samples. J. Appl. Physiol. 28, 234–236 5413312 10.1152/jappl.1970.28.2.234

[b31-0071185] McMinn J. Wei M. Schupf N. Cusmai J. Johnson E. B. Smith A. C. Weksberg R. Thaker H. M. Tycko B. (2006). Unbalanced placental expression of imprinted genes in human intrauterine growth restriction. Placenta 27, 540–549 16125225 10.1016/j.placenta.2005.07.004

[b32-0071185] Oh-McGinnis R. Bogutz A. B. Lefebvre L. (2011). Partial loss of Ascl2 function affects all three layers of the mature placenta and causes intrauterine growth restriction. Dev. Biol. 351, 277–286 21238448 10.1016/j.ydbio.2011.01.008 PMC3820550

[b33-0071185] Qian N. Frank D. O’Keefe D. Dao D. Zhao L. Yuan L. Wang Q. Keating M. Walsh C. Tycko B. (1997). The IPL gene on chromosome 11p15.5 is imprinted in humans and mice and is similar to TDAG51, implicated in Fas expression and apoptosis. Hum. Mol. Genet. 6, 2021–2029 9328465 10.1093/hmg/6.12.2021

[b34-0071185] Saenger P. Czernichow P. Hughes I. Reiter E. O. (2007). Small for gestational age: short stature and beyond. Endocr. Rev. 28, 219–251 17322454 10.1210/er.2006-0039

[b35-0071185] Salas M. John R. Saxena A. Barton S. Frank D. Fitzpatrick G. Higgins M. J. Tycko B. (2004). Placental growth retardation due to loss of imprinting of Phlda2. Mech. Dev. 121, 1199–1210 15327781 10.1016/j.mod.2004.05.017

[b36-0071185] Schmittgen T. D. Livak K. J. (2008). Analyzing real-time PCR data by the comparative C(T) method. Nat. Protoc. 3, 1101–1108 18546601 10.1038/nprot.2008.73

[b37-0071185] Shukla P. K. Sittig L. J. Ullmann T. M. Redei E. E. (2011). Candidate placental biomarkers for intrauterine alcohol exposure. Alcohol. Clin. Exp. Res. 35, 559–565 21143252 10.1111/j.1530-0277.2010.01373.x PMC3117908

[b38-0071185] Tunster S. J. Tycko B. John R. M. (2010). The imprinted Phlda2 gene regulates extraembryonic energy stores. Mol. Cell. Biol. 30, 295–306 19884348 10.1128/MCB.00662-09 PMC2798284

[b39-0071185] Tunster S. J. Van de Pette M. John R. M. (2012). Impact of genetic background on placental glycogen storage in mice. Placenta 33, 124–127 22153913 10.1016/j.placenta.2011.11.011

[b40-0071185] Tunster S. J. Jensen A. B. John R. M. (2013). Imprinted genes in mouse placental development and the regulation of fetal energy stores. Reproduction 145, R117–R137 23445556 10.1530/REP-12-0511

[b41-0071185] Valero De Bernabé J. Soriano T. Albaladejo R. Juarranz M. Calle M. E. Martínez D. Domínguez-Rojas V. (2004). Risk factors for low birth weight: a review. Eur. J. Obstet. Gynecol. Reprod. Biol. 116, 3–15 15294360 10.1016/j.ejogrb.2004.03.007

[b42-0071185] Withington S. L. Scott A. N. Saunders D. N. Lopes Floro K. Preis J. I. Michalicek J. Maclean K. Sparrow D. B. Barbera J. P. Dunwoodie S. L. (2006). Loss of Cited2 affects trophoblast formation and vascularization of the mouse placenta. Dev. Biol. 294, 67–82 16579983 10.1016/j.ydbio.2006.02.025

[b43-0071185] Witt W. P. Wisk L. E. Cheng E. R. Hampton J. M. Hagen E. W. (2012). Preconception mental health predicts pregnancy complications and adverse birth outcomes: a national population-based study. Matern. Child Health J. 16, 1525–1541 22124801 10.1007/s10995-011-0916-4 PMC3605892

[b44-0071185] Zheng-Fischhöfer Q. Kibschull M. Schnichels M. Kretz M. Petrasch-Parwez E. Strotmann J. Reucher H. Lynn B. D. Nagy J. I. Lye S. J. (2007). Characterization of connexin31.1-deficient mice reveals impaired placental development. Dev. Biol. 312, 258–271 17961533 10.1016/j.ydbio.2007.09.025

