# Supplementary material for: Isolating the role of elevated Phlda2 in asymmetric late fetal growth restriction in mice
Source: Dis Model Mech. 2014 Aug 1;7(10):1185–91. doi: 10.1242/dmm.017079 (PMC4174529; doi:10.1242/dmm.017079)
Supplement: Supplementary Material [file supp_7_10_1185__index.html]

Supplementary Material 

# Isolating the role of elevated *Phlda2* in asymmetric late fetal growth restriction in mice

## DMM017079 Supplementary Material

**Files in this Data Supplement:**

- **Supplementary Material**
