# Supplementary material for: Isolating the role of elevated Phlda2 in asymmetric late fetal growth restriction in mice
Source: Dis Model Mech. 2014 Aug 1;7(10):1185–91. doi: 10.1242/dmm.017079 (PMC4174529; doi:10.1242/dmm.017079)
Supplement: Supplementary Material [file supp_017079_DMM017079.pdf]

Supplemental data for figure 1-5

Figure 1

A: E14.5 Transgene Expression Levels Relative to WT			
	Phlda2		*Slc22a18*
$Phlda2^{+/+BACx1(129)}$	1.80 ± 0.071<br>(p = 0.0239)		2.17 ± 0.074<br>(p = 2.34 x 10^{-4})
B: Fetal Weights			
	E14.5	E16.5	E18.5
$Phlda2^{+/+(129)}$	216.7 mg ± 2.7<br>n = 48	560.3 mg ± 9.0<br>n = 25	1184.4 mg ± 8.7<br>n = 36
$Phlda2^{+/+BACx1(129)}$	212.6 mg ± 4.3<br>n = 43	534.7 mg ± 12.5<br>n = 24	1073.8 mg ± 18.5<br>n = 48
Ratio and p value	98.1%<br>p = 0.402	95.4%<br>p = 0.101	90.7%<br>p = 5.33 x 10^{-6}
C: Placenta Weights			
$Phlda2^{+/+(129)}$	75.4 mg ± 0.8<br>n = 48	76.8 mg ± 0.9<br>n = 25	75.1 mg ± 1.2<br>n = 36
$Phlda2^{+/+BACx1(129)}$	66.4 mg ± 1.0<br>n = 43	63.6 mg ± 1.5<br>n = 24	67.4 mg ± 1.1<br>n = 48
Ratio and p value	88.1%<br>p = 1.76 x 10^{-10}	82.7%<br>p = 3.53 x 10^{-10}	89.7%<br>p = 9.74 x 10^{-6}
$\chi 2$ Test. p = 0.05; Degrees of Freedom = 1; Critical Value = 3.841			
$\chi 2$	0.275	0.020	1.714

Figure 2

A: E14.5 Transgene Expression Levels Relative to WT			
	Phlda2		*Slc22a18*
$Phlda2^{-/+BACx1}$	1.15 ± 0.03<br>(p = 0.546)		1.92 ± 0.06<br>(p = 4.75 x 10^{-4})
B: E18.5 Fetal Weights			
$Phlda2^{+/+}$	$Phlda2^{+/+BACx1}$	$Phlda2^{-/+}$	$Phlda2^{-/+BACx1}$
1123.3 mg ± 13.8<br>n = 32	1038.7 mg ± 23.4<br>n = 16	1116.1 mg ± 19.1<br>n = 26	1144.3 mg ± 23.4<br>n = 18
-	92.5%<br>p = 0.00180	99.4%<br>p = 0.753	101.9%<br>p = 0.415
C: E18.5 Placenta Weights			
71.5 mg ± 0.9<br>n = 32	63.6 mg ± 2.0<br>n = 16	94.9 mg ± 4.1<br>n = 26	68.0 mg ± 2.2<br>n = 18
-	89%<br>p = 1.83 x 10^{-4}	132.8%<br>p = 8.41 x 10^{-8}	95.2%<br>p = 0.102

Figure 3

A: Brain Proportion of Body Weight			
$Phlda2^{+/+}$	$Phlda2^{+/+BACx1}$	$Phlda2^{-/+}$	$Phlda2^{-/+BACx1}$
5.26% ± 0.10<br>n = 19	5.58% ± 0.06<br>n = 15	5.22% ± 0.19<br>n = 17	5.23% ± 0.07<br>n = 15

-	105.9%<br>p = 0.0202	99.2%<br>p = 0.849	99.3%<br>p = 0.773
A: Lungs Proportion of Body Weight			
Phlda2$^{+/+}$	*Phlda2*$^{+/+BACx1}$	*Phlda2*$^{-/+}$	*Phlda2*$^{-/+BACx1}$
3.98% ± 0.13<br>n = 19	3.99% ± 0.14<br>n = 15	3.96% ± 0.14<br>n = 17	3.77% ± 0.15<br>n = 15
-	100.2%<br>p = 0.960	99.3%<br>p = 0.887	94.7%<br>p = 0.291
A: Heart Proportion of Body Weight			
Phlda2$^{+/+}$	*Phlda2*$^{+/+BACx1}$	*Phlda2*$^{-/+}$	*Phlda2*$^{-/+BACx1}$
0.76% ± 0.034<br>n = 19	0.77% ± 0.032<br>n = 15	0.73% ± 0.025<br>n = 17	0.74% ± 0.042<br>n = 15
-	101.4%<br>p = 0.819	96.1%<br>p = 0.493	97.0%<br>p = 0.667
A: Liver Proportion of Body Weight			
Phlda2$^{+/+}$	*Phlda2*$^{+/+BACx1}$	*Phlda2*$^{-/+}$	*Phlda2*$^{-/+BACx1}$
6.83% ± 0.17<br>n = 19	6.12% ± 0.20<br>n = 15	6.73% ± 0.27<br>n = 17	6.32% ± 0.18<br>n = 15
-	89.7%<br>p = 0.0119	98.6%<br>p = 0.757	92.6%<br>p = 0.0500
A: Kidney Proportion of Body Weight			
Phlda2$^{+/+}$	*Phlda2*$^{+/+BACx1}$	*Phlda2*$^{-/+}$	*Phlda2*$^{-/+BACx1}$
0.43% ± 0.012<br>n = 38	0.39% ± 0.013<br>n = 30	0.43% ± 0.009<br>n = 34	0.41% ± 0.015<br>n = 30
-	90.5%<br>p = 0.0225	99.9%<br>p = 0.986	94.9%<br>p = 0.251

B: Brain Proportion of Body Weight	
Phlda2$^{+/+}$	*Phlda2*$^{+/+BACx3}$
3.65% ± 0.069<br>n = 28	3.89 ± 0.070<br>n = 27
-	106.6%<br>p = 0.0170

C: Weight					
	E14.5	E16.5	E18.5	P0	P1
Phlda2$^{+/+}$	222 mg ± 2.9<br>n = 47	600.0 mg ± 8.8<br>n = 30	1.24 g ± 0.08<br>n = 57	1.41 g ± 0.009<br>n = 79	1.62 g ± 0.021<br>n = 26
Phlda2$^{+/+}$ BACx3	218.8 mg ± 4.5<br>n = 38	560.5 mg ± 7.6<br>n = 28	1.08 g ± 0.013<br>n = 38	1.25 g ± 0.014<br>n = 75	1.51 g ± 0.023<br>n = 23
Ratio and p value	98.5%<br>p = 0.508	93.4%<br>p = 0.00139	93.4%<br>p = 7.60 x 10^{-19}	88.7%<br>p = 3.29 x 10^{-17}	92.7%<br>p = 3.87 x 10^{-4}
	P7	P14	P21	P28	P21 (BACx1)
Phlda2$^{+/+}$	3.51 g ± 0.078<br>n = 54	6.45 g ± 0.100<br>n = 78	8.06 g ± 0.159<br>n = 78	12.71 g ± 0.261<br>n = 76	
Phlda2$^{+/+}$ BACx3	3.35 g ± 0.082	6.45 g ± 0.106<br>n = 69	8.09 g ± 0.161<br>n = 69	12.86 g ± 0.241	

	n = 23			n = 68	
Ratio and p value	95.3% *p* = 0.147	99.9% *p* = 0.973	100.4% *p* = 0.889	101.2% *p* = 0.675	

Figure 4

A: Fetal Weights (BL6)			
	E14.5	E16.5	E18.5
Phlda2$^{+/+}$	236.1 mg ± 2.3 n = 57	594.6 mg ± 6.3 n = 52	1138.5 mg ± 9.1 n = 48
Phlda2$^{+/+BACx1}$	243.5 mg ± 6.9 n = 24	587.3 mg ± 9.1 n = 27	1149.1 mg ± 23.6 n = 17
Ratio and p value	103.1% *p* = 0.198	98.8% *p* = 0.468	100.9% *p* = 0.610
Phlda2$^{+/+BACx3}$	227.9 mg ± 5.5 n = 22	567.1 mg ± 11.4 n = 21	1163.2 mg ± 27.7 n = 24
Ratio and p value	96.5% *p* = 0.108	95.4% *p* = 0.0281	102.2% *p* = 0.294
B: Placenta Weights BL6			
Phlda2$^{+/+}$	103.6 mg ± 1.3 n = 57	103.5 mg ± 1.5 n = 52	96.6 mg ± 1.4 n = 48
Phlda2$^{+/+BACx1}$	88.4 mg ± 2.2 n = 24	88.7 mg ± 1.6 n = 27	87.8 mg ± 1.8 n = 17
Ratio and p value	85.3% $p = 1.44 \times 10^{-8}$	85.7% $p = 1.80 \times 10^{-8}$	90.8% $p = 8.63 \times 10^{-4}$
Phlda2$^{+/+BACx3}$	89.9 mg ± 2.1 n = 22	85.0 mg ± 2.0 n = 21	89.6 mg ± 2.7 n = 24
Ratio and p value	86.7% $p = 2.57 \times 10^{-7}$	85.0% $p = 3.10 \times 10^{-7}$	92.8% *p* = 0.0116

C: F:P Ratios						
	E14.5		E16.5		E18.5	
	BL6	129	BL6	129	BL6	129
Phlda2$^{+/+}$	2.29 ± 0.030 n = 57	2.83 ± 0.030 n = 95	5.78 ± 0.074 n = 52	7.32 ± 0.095 n = 55	11.89 ± 0.19 n = 48	15.96 ± 0.16 n = 93
Phlda2$^{+/+BACx1}$	2.75 ± 0.037 n = 24	3.15 ± 0.062 n = 43	6.66 ± 0.138 n = 27	8.49 ± 0.255 n = 24	13.14 ± 0.28 n = 17	16.11 ± 0.32 n = 48
Ratio and p value	120.1% $p = 1.83 \times 10^{-13}$	111.3% $p = 5.01 \times 10^{-7}$	115.2% $p = 1.13 \times 10^{-8}$	116.1% $p = 9.55 \times 10^{-7}$	110.5% $p = 8.49 \times 10^{-4}$	100.9% *p* = 0.647
Phlda2$^{+/+BACx3}$	2.55 ± 0.064 n = 22	3.40 ± 0.087 n = 38	6.47 ± 0.129 n = 21	8.31 ± 0.204 n = 28	13.21 ± 0.43 n = 24	15.67 ± 0.34 n = 38
Ratio and p value	111.4% $p = 7.39 \times 10^{-5}$	120.3% $p = 8.81 \times 10^{-13}$	111.9% $p = 7.29 \times 10^{-6}$	113.6 $p = 2.79 \times 10^{-6}$	111.1% $p = 1.67 \times 10^{-3}$	98.2 *p* = 0.369

Figure 5

B: Placental Glycogen (mg)				
	E14.5 (BACx1)	E16.5 (BACx1)	E18.5 (BACx1)	E18.5 (BACx3)
Phlda2$^{+/+(129)}$	0.422 mg ±0.037<br>n = 19	0.235 mg ±<br>0.025<br>n = 25	0.155 mg ±<br>0.014<br>n = 35	0.183 mg ±<br>0.011<br>n = 24
Phlda2$^{+/+BAC(129)}$	0.189 mg ±<br>0.010<br>n = 21	0.101 mg ±<br>0.010<br>n = 24	0.073 mg ±<br>0.004<br>n = 47	0.061 ± 0.004<br>n = 12
Ratio and p value	44.8%<br>$p = 1.92 \times 10^{-7}$	43.2%<br>$p = 1.36 \times 10^{-5}$	47.0%<br>$p = 9.42 \times 10^{-9}$	33.5%<br>$p = 2.02 \times 10^{-9}$
C: Placental Glycogen (mg)				
Phlda2$^{+/+(BL6)}$	1.423 mg ±<br>0.156<br>n = 10	1.373 mg ±<br>0.124<br>n = 18	0.938 mg ±<br>0.101<br>n = 17	0.819 mg ±<br>0.059<br>n = 14
Phlda2$^{+/+BAC(BL6)}$	0.888 mg ±<br>0.070<br>n =	0.779 mg ±<br>0.050<br>n = 20	0.511 mg ±<br>0.028<br>n = 12	0.472 mg ±<br>0.028<br>n = 16
Ratio and p value	62.4%<br>$p = 1.44 \times 10^{-3}$	56.7%<br>$p = 4.95 \times 10^{-5}$	54.5%<br>$p = 1.78 \times 10^{-3}$	57.6%<br>$p = 6.16 \times 10^{-6}$
D: Placental Glycogen (mg/g)				
Phlda2$^{+/+(129)}$	5.48 mg/g ±<br>0.39<br>n = 19	3.08 mg/g ±<br>0.33<br>n = 25	2.06 mg/g ±<br>0.18<br>n = 35	2.42 mg ± 0.13<br>n = 24
Phlda2$^{+/+BAC(129)}$	2.90 mg/g ±<br>0.14<br>n = 21	1.59 mg/g ±<br>0.15<br>n = 0.15	1.08 mg/g ±<br>0.05<br>n = 47	0.98 mg ± 0.05<br>n = 12
Ratio and p value	53.0%<br>$p = 1.59 \times 10^{-7}$	51.7%<br>$p = 1.77 \times 10^{-4}$	52.2%<br>$p = 1.16 \times 10^{-7}$	40.4%<br>$p = 2.30 \times 10^{-9}$
E: Placental Glycogen (mg/g)				
Phlda2$^{+/+(BL6)}$	14.35 mg/g ±<br>1.10<br>n = 10	13.04 mg/g ±<br>0.96<br>n = 18	9.66 mg/g ±<br>0.62<br>n = 17	8.76 mg ± 0.68<br>n = 14
Phlda2$^{+/+BAC(BL6)}$	9.81 mg/g ±<br>0.64<br>n = 17	9.06 mg/g ±<br>0.60<br>n = 20	5.91 mg/g ±<br>0.29<br>n =12	5.17 mg ± 0.25<br>n = 16
Ratio and p value	68.4%<br>$p = 7.34 \times 10^{-4}$	69.5%<br>$p = 1.00 \times 10^{-3}$	61.2%<br>$p = 5.17 \times 10^{-5}$	59.0%<br>$p = 1.46 \times 10^{-5}$
F: E18.5 Placental Glycogen (mg)				
	129	BL6	Ratio and p value (vs *Phlda2*$^{+/+(129)}$)	
Phlda2$^{+/+}$	0.167 mg ±<br>0.0094<br>n = 59	0.884 mg ±<br>0.061<br>n = 31	529.3%<br>$p = 6.07 \times 10^{-27}$	
Phlda2$^{+/+BACx1}$	0.073 mg ±<br>0.0038<br>n = 47	0.511 mg ±<br>0.028<br>n = 12	306.2%<br>$p = 5.99 \times 10^{-22}$	
Phlda2$^{+/+BACx3}$	0.061 mg ±<br>0.0040<br>n = 12	0.472 mg ±<br>0.028<br>n = 16	282.4%<br>$p = 5.98 \times 10^{-21}$	
